# Endoscopic and surgical treatment of necrotizing pancreatitis—a comparison of short- and long-term outcome

**DOI:** 10.1007/s00423-024-03244-9

**Published:** 2024-02-12

**Authors:** Lea Timmermann, Svenja Schönauer, Karl Herbert Hillebrandt, Matthäus Felsenstein, Johann Pratschke, Thomas Malinka, Christian Jürgensen

**Affiliations:** 1 Department of Surgery, Charité – Universitätsmedizin Berlin, corporate member of Freie Universität Berlin, Humboldt-Universität zu Berlin, and Berlin Institute of Health, Berlin, Germany; 2 Department of Hepatology and Gastroenterology, Charité – Universitätsmedizin Berlin, corporate member of Freie Universität Berlin, Humboldt-Universität zu Berlin, and Berlin Institute of Health, Berlin, Germany

**Keywords:** Necrotizing pancreatitis, Endoscopic necrosectomy, Peri-interventional morbidity and mortality

## Abstract

**Background:**

Acute necrotizing pancreatitis is still related to high morbidity and mortality rates. Minimal-invasive treatment options, such as endoscopic necrosectomy, may decrease peri-interventional morbidity and mortality. This study aims to compare the initial operative with endoscopic treatment on long-term parameters, such as endocrine and exocrine functionality, as well as mortality and recurrence rates.

**Methods:**

We included 114 patients, of whom 69 were treated with initial endoscopy and 45 by initial surgery. Both groups were further assessed for peri-interventional and long-term parameters.

**Results:**

In the post-interventional phase, patients in the group of initial surgical treatment (IST) showed significantly higher rates of renal insufficiency (*p* < 0.001) and dependency on invasive ventilation (*p* < 0.001). The in-house mortality was higher in the surgical group, with 22% vs. 10.1% in the group of patients following initial endoscopic treatment (IET; *p* = 0.077). In long-term follow-up, the overall mortality was 45% for IST and 31.3% for IET (*p* = 0.156). The overall in-hospital stay and intensive care unit (ICU) stay were significantly shorter after IET (*p* < 0.001). In long-term follow-up, the prevalence of endocrine insufficiency was 50% after IST and 61.7% after IET (*p* = 0.281). 57.1% of the patients following IST and 16.4% of the patients following IET had persistent exocrine insufficiency at that point (*p* =  < 0.001). 8.9% of the IET and 27.6% of the IST patients showed recurrence of acute pancreatitis (*p* = 0.023) in the long-term phase.

**Conclusion:**

In our cohort, an endoscopic step-up approach led to a reduced in-hospital stay and peri-interventional morbidity. The endocrine function appeared comparable in both groups, whereas the exocrine insufficiency seemed to recover in the endoscopic group in the long-term phase. These findings advocate for a preference for endoscopic treatment of acute necrotizing pancreatitis whenever feasible.

## Introduction

Acute necrotizing pancreatitis remains a life-threatening disease with mortality rates as high as 20–30% [1, 2]. Around 20% of the patients suffering from acute pancreatitis, an increasing health issue [3, 4], develop this severe progressive form [5, 6]. The main aetiologies of acute pancreatitis in developed states remain biliary and alcohol abuse [7]. However, post-interventional pancreatitis following interventions such as endoscopic retrograde cholangiopancreaticography (ERCP) gains more importance. Alongside belt-shaped abdominal pain, the consequences of the fluid shift from intra- to extravascular compartments, e.g., hypotonia, renal insufficiency, and respiratory insufficiency, dominate the early course of severe acute pancreatitis [8], demanding close-knit fluid replacement and clinical observation.

Open abdominal surgery, including necrosectomy, has been the treatment standard for necrotizing pancreatitis for decades. In the last years, minimal-invasive techniques, including laparoscopy, retroperitoneoscopy, interventional radiology, and endoscopy, rapidly evolved and a step-up approach was established. This, however, requires an interdisciplinary consensus and treatment plan and currently favors endoscopic over more invasive treatment options to minimize tissue trauma and negative side effects of surgery. Nevertheless, there are still a few indications requiring immediate surgical therapy. Abdominal compartment syndrome, intestinal perforation, and severe bleeding, therefore, remain emergency indications for immediate surgical therapy. Apart from emergency indications for immediate surgical therapy, the advantages of a step-up approach are clear at hand. The implementation of a step-up approach has been described to effectively decrease mortality and peri-interventional morbidity. van Brunschot et al., however, showed comparable rates for major complications in an endoscopic and surgical approach and a mortality rate of 18% for their endoscopic approach in a prospective multicentre study [9].

Alongside invasiveness of a treatment strategy, the exact timing and furthermore the right indication for—even minimally—invasive treatment are crucial. In the past, immediate surgical necrosectomy was performed, but several studies showed that early surgery in stable patients with sterile necrosis increases mortality [10]. Conservative management for sterile necroses is associated with mortality rates as low as 2% [11]. The occurrence of infection, however, can rapidly deteriorate clinical presentation and may enforce prompt interventions [10]. Other studies showed that even bacterial translocation and consecutive bacteremia do not necessarily deteriorate the clinical course and outcome [12]. For sterile necrosis, only persistent symptoms indicate the quest for interventional therapy. Infected necrosis also poses an indication for an intervention, which is recommended to be postponed during the first phase of acute pancreatitis, at least two, better 4 weeks in stable patients, in order to decrease mortality [13]. Adequate liquification and encapsulation after this period increase the probability of complete necrosectomy. Apart from early-phase morbidity and mortality, there is a lack of evidence regarding the long-term outcome in patients following surgical or endoscopic necrosectomy. The objective of this study was to compare long-term results of patients undergoing initial surgery with those undergoing initial endoscopic treatment with emphasis on endocrine and exocrine function as well as recurrence rates and survival.

## Methods

Data of all consecutive patients undergoing initial endoscopic therapy (IET) for necrotizing pancreatitis from 2002 to 2014 performed by the same consulting gastroenterologist (CJ) were identified and their data were further reviewed. This cohort was compared to data of all consecutive patients undergoing initial surgical therapy (IST) in our tertiary referral center for pancreatic surgery in the same period for the indication of necrotizing pancreatitis. Decisive for the definition of both groups was the initial approach for necrosectomy even if later on surgical treatment became necessary after IET or the other way round. The decision for or against IST or IET was made according to a step-up approach from minimally invasive to invasive always favoring an endoscopic approach if applicable. IST was indicated cautiously for certain reasons further described in the following. We excluded all patients under age, patients developing a postoperative necrotizing pancreatitis of the pancreatic remnant following (elective) pancreatic surgery and patients developing acute necrotizing pancreatitis following pancreas transplantation. We excluded all patients who did not undergo any type of intervention for the necrosis except for percutaneous drainage (e.g., CT-guided) and conservative treatment.

The study was conducted according to the Declaration of Helsinki guidelines and approved by the Institutional Review Board of Charité Universitätsmedizin Berlin (protocol code; EA2/035/14). According to the Equator network decision tree, no reporting guideline is applicable to our study.

### Admission and treatment

Patients with acute necrotizing pancreatitis included in this study were primarily admitted to our centre in an emergency setting via one of our emergency departments or directly to a high-dependency unit. In a few cases of post-ERCP pancreatitis, patients were already in an inpatient setting in our clinic. Initial emergency assessment is standardized and includes a physical examination, continuous non-invasive measurement of vital signs, and focused abdominal sonography. The time of further imaging (computed tomography) was scheduled based on each patient’s condition individually. At the time of initial admission, the necrotizing aspect was mostly unapparent, if patients were admitted directly to our clinic. An interdisciplinary consent of attending gastroenterologists and visceral surgeons individually determines the further course. CT morphological signs for intestinal perforation and abdominal compartment syndrome indicate primary and prompt surgical intervention. All other patients were admitted to high-dependency care or a surgical or gastroenterological ward dependent on their clinical state and stability of vital signs or signs of organ failure. Crystalloid fluids were administered close-knit and clinically adapted as demanded. Additional conservative measures were administered depending on organ function, e.g., dialysis, (non)invasive ventilation, and administration of vasopressors. Necrosectomy was scheduled dependent on clinical status and CT morphological liquification of necrosis in the further course of hospitalization.

### Endoscopic treatment

Every patient was treated according to a step-up approach from minimally invasive to invasive. Therefore, every patient not requiring emergency surgery (e.g., intestinal perforation, abdominal compartment syndrome, or severe erosion with no angiographic treatment option) with endoscopically accessible necrosis was selected for IET. If indicated in an interdisciplinary consent, endoscopic necrosectomy was planned according to pre-interventional computed tomography requiring both adequate demarcation and anatomical proximity to the dorsal gastric or duodenal wall. It usually consists of a three-step approach. In the first step, endosonography correlates the pre-interventional scans and indicates the optimal drainage site. With guidance of endosonography, the necrosis is then punctured with a 19-G needle (e.g., Echotip®; Wilson-Cook Co, Winston-Salem, NC, USA) and transgastric pigtail drainages (e.g., 10-Fr Gastrosoft biliary endoprosthesis®; OptiMed, Ettlingen, Germany) are inserted after the drainage site is dilated with a balloon catheter (e.g., CRE®; Boston Scientific, Microvasive, Cork, Ireland). After the inlaying drainages consolidated the drainage site for 2 to 3 days, the second step, the necrosectomy itself, is performed. This step is repeated until no more accessible necrosis is left. In between the sessions, transgastric pigtail drainages are repeatedly inserted, and liquefied necrosis can internally drain through the stomach and intestines. The third step describes the (final) consolidation process after necrosectomy is finished and persists of the insertion of transgastric pigtail drainages that will usually be removed after six more weeks of consolidation in an outpatient setting.

### Surgical treatment

In cases of intestinal perforation, abdominal compartment syndrome, or severe erosion with no angiographic treatment option, the emergency setup dictates invasiveness and access to the abdominal cavity mainly via median explorative laparotomy. Apart from emergencies, the main indication for operative treatment was uncontrolled infection or symptoms related to side effects of the necrosis such as intestinal obstruction or an endoscopically inaccessible localization.

### Parameters

We included the following parameters: age, sex, cause of pancreatitis, organ failure at the time of diagnosis, sepsis at the time of diagnosis, pre-existing diabetes mellitus, pre-existing exocrine insufficiency of the pancreas, in-hospital stay, intensive care unit stay, hospital readmission, post-interventional organ failure, recurrence of pancreatitis in long course and mortality. The state of diabetes mellitus with or without insulin dependency and the exocrine insufficiency were reviewed for the short and long term. Worsening of the exocrine insufficiency was defined according to the necessity of medication (enzyme substitution) at each point of evaluation. Worsening of the endocrine function was evaluated according to the necessity of medication or the need for intensifying. The initial necessity of oral medication turning into insulin dependency is an example. To minimize biases, the pre-interventional state was defined as the state prior to hospital admission and systemic inflammation as peri-interventional fasting and parenteral nutrition certainly influence e.g. the necessity of insulin administration. The analysis is divided into short-term and long-term outcome. The short-term outcome is defined as parameter and data collected during the initial inpatient stay. The long-term follow-up 1 consisted of a clinical review of the patients regarding exocrine and endocrine function as well as recurrence after 6 months. Long-term follow-up 2 was defined as the date of the latest contact and included a clinical review regarding exocrine and endocrine insufficiency and recurrence. The mean follow-up period following IST was 5.3 years (SD 3.8). The mean follow-up period following IET was 4 years (SD 3). Long-term data was collected from outpatient visits and did not follow a certain follow-up protocol.

### Statistics

All data were processed using SPSS version 27.0 (IBM, Armonk, NY, USA). Two-tailed Pearson’s chi-square test was performed on categorical and ordinal scaled data, and Student’s *t*-test and Mann-Whitney *U* test were performed on interval scaled data. The Breslow-Day test was performed alongside Kaplan-Meier curves for survival functions. Significance tests were two-sided, and *p* < 0.05 was considered statistically significant.

## Results

### Patient’s characteristics and pre-interventional course

An overall of 114 patients was included in this study. Sixty-nine of them underwent IET, and 45 underwent IST due to necrotizing pancreatitis. The median interval between initial diagnosis and first necrosectomy was 44 days in the IST group and 38.5 days in the IET group (*p* = 0.883). In the IST group, the most common technique was selective necrosectomy in 73% of the cases, followed by pancreatic head resection in 15.5% of the cases, distal pancreatectomy in 8.9% of the cases, and total pancreatectomy in 2.2% of the cases. In the IET group, necrosectomy was most commonly performed via the dorsal gastric wall in 92.8% of the cases, followed by transduodenal necrosectomy in 5.8% of the cases. Both methods were combined in 1.4% of the cases. The mean follow-up period following IST was 5.3 years (SD 3.8 years), and for IET 3.9 years (SD 3.0 years). The most common cause of pancreatitis in the IET group was biliary. In the IST group, the most common cause was alcohol. Patients following IST were significantly younger, with a median of 57 years, compared to a median of 64 following IET (*p* = 0.002). Regarding pre-interventional endocrine insufficiency, patients from the IST and IET group were comparable (*p* = 0.784). None of the patients in the IET group (0%) and two patients from the IET group (4.8%) had pre-existing exocrine insufficiency, which did not appear to be statistically significant (*p* = 0.069). Table 1 indicates the patient’s characteristics and pre-interventional findings.

**Table 1:** Patients’ characteristics and pre-interventional parameters; baseline characteristics of patients undergoing either initial surgery or endoscopy

Characteristics *N* (%)	All patients (*N* = 114)	Endoscopy (*N* = 69)	Surgery (*N* = 45)	*p*-value
Sex, *N* (%)				0.508
Male	80 (70.2)	50 (72.5)	30 (66.6)	
Female	34 (29.8)	19 (27.5)	15 (33.4)	
Age (years)				***0.002***
Median	60.5	64	57	
Minimum	21	21	23	
Maximum	87	87	82	
Cause of pancreatitis, *N* (%)				***0.036***
Biliary	42 (36.8)	32 (46.4)	10 (22.7)	
Alcohol	33 (28.9)	19 (27.5)	14 (31.8)	
Post**-**interventional	15 (13.2)	4 (5.8)	11 (25)	
Toxic	2 (1.8)	1 (1.4)	1 (2.3)	
Unknown/other	21 (18.3)	13 (18.8)	8 (18.2)	
Pre-existing diabetes mellitus, *N* (%)	27 (23.7)	17 (25)	10 (22.7)	0.784
Pre-existing exocrine insufficiency, *N* (%)	2 (1.8)	0 (0)	2 (4.8)	0.069
Pre-interventional sepsis, *N* (%)	29 (30.9)	14 (28.0)	15 (34.1)	0.523
Pre-interventional multi-organ failure, *N* (%)	24 (21.1)	16 (23.5)	8 (18.2)	0.501
Pre-interventional invasive ventilation, *N* (%)	38 (33.6)	17 (24.6)	21 (47.7)	***0.011***
Pre-interventional acute renal insufficiency, *N* (%)	36 (31.9)	20 (29)	16 (36.4)	0.412
Pre-interventional dialysis	8 (7.0)	5 (7.2)	3 (6.7)	0.906

Bold-italic was used to indicate singificant *p*-values

Both groups furthermore did not significantly differ regarding pre-interventional organ failure and sepsis. Patients following IST were significantly more likely to undergo invasive ventilation compared to IET before the first intervention (*p* = 0.011). Indications that finally led to a surgical approach in the step-up process were intestinal perforation in two cases (4.5%), severe bleeding in three patients (6.8%), suspected underlying malignancy in one case (2.3%), and clinical deterioration in 17 cases (38.6%). In the other cases, the localization of necrosis inaccessible for endoscopic treatment (e.g., paracolic gutter) led to the surgical approach.

### Short-term outcome and in-hospital stay

Following IST, a mean of 2.1 operations was performed, whereas in the IET group, a mean of 6.0 procedures was performed (*p* < 0.001). In the IST group, mainly lavages and changes of the abdominal dressings were performed. The latter procedures in the IET group included endoscopic access, endoscopic necrosectomy sessions, and removal of endoscopic drainages. Table 2 indicates data from the post-interventional courses.

**Table 2:** In-hospital stay comparing peri-interventional complications and outcome parameters of patients either undergoing initial surgery or endoscopy

Characteristics *N* (%)	All patients (*N* = 114)	Endoscopy (*N* = 69)	Surgery (*N* = 45)	*p*-value
Overall in-hospital stay (days)				***< 0.001***
Median	34	29	74	
Minimum	5	5	14	
Maximum	343	142	343	
Post-interventional in-hospital stay (days)				***0.002***
Median	27	23	60	
Minimum	2	4	2	
Maximum	332	118	332	
Overall ICU stay				***< 0.001***
Median	16	1	26.5	
Minimum	0	0	0	
Maximum	190	88	190	
Post-interventional ICU stay (days)				***< 0.001***
Median	8	1	16.5	
Minimum	0	0	0	
Maximum	183	86	183	
Reoperation/unplanned surgical intervention	24 (21.1)	3 (4.5)	21 (46.7)	*** < 0.001***
Post-interventional sepsis, *N* (%)	18 (15.4)	7 (18.9)	11 (25)	0.512
Post-interventional multi-organ failure, *N* (%)	11 (11.5)	3 (5.8)	8 (18.2)	0.057
Post-interventional newly implemented invasive ventilation, *N* (%)	8 (7.0)	3 (5.8)	5 (11.4)	*** < 0.001***
Post-interventional new kidney failure, *N* (%)	16 (16.7)	5 (9.6)	11 (25.0)	***< 0.001***
Post-interventional new necessity of dialysis, *N* (%)	4 (3.5)	2 (2.8)	2 (4.5)	0.869
In-house mortality, *N* (%)	17 (14.9)	7 (10.1)	10 (22.2)	0.077

Bold-italic was used to indicate singificant *p*-values

The overall in-hospital stay (*p* < 0.001), post-interventional in-hospital stay (*p* = 0.002), overall ICU stay (*p* < 0.001), and post-interventional ICU stay (*p* < 0.001) were all significantly longer following IST. The rate of further unplanned reoperations was also significantly higher following IST, with 46.7% compared to the need of post-interventional surgery following IET in 4.5% of the cases (*p* < 0.001). Leading causes for reoperation apart from planned re-looks following IST were intestinal perforation in four cases (20.0%), intra-abdominal abscesses in four cases (20.0%), bleeding in four cases (20.0%), insufficiency of enteric anastomoses in two cases (10.0%), biliary leakage following cholecystectomy in two cases (10.0%), wound infection in two cases (10.0%), intestinal ischemia in one case (5.0%), and persisting peritonitis in two cases (10.0%). Following IET, one patient underwent surgery due to bleeding (33.3%), one due to intestinal obstruction and ileus (33.3%), and one due to an abscess (33.3%). Seven patients following IET (18.9%) and 11 patients following IST (25%) fit the criteria for post-interventional sepsis (*p* = 0.512). Three patients in the endoscopic group (5.8%) and eight patients in the IST group (18.2%) met the criteria for post-interventional multi-organ failure. This finding did not appear statistically significant (*p* = 0.057). Regarding post-interventional newly implemented invasive ventilation, both groups differed significantly (*p* < 0.001). It was necessary in the IET group in three (5.8%) of the patients and in the IST group in five (11.4%) of the patients. The rate of post-interventional kidney failure was significantly higher in the IST group with 25% compared to 9.6% in the IET group (*p* < 0.001). However, the necessity or dialysis was comparable in both groups (*p* = 0.869).

### Diabetes mellitus and exocrine insufficiency

Following the interventions, 46.3% of the patients after IST showed a worsening of the diabetes mellitus level either by new dependency on insulin or new manifestation. In the IET group, 32.8% showed further deterioration of their diabetes mellitus levels (necessity for oral antidiabetic medication or insulin; *p* = 0.012). Regarding the exocrine function, patients after IST showed a worsening in 32.4% of the cases, whereas in the IET group, it was in 16.7% of the cases (*p* = 0.065). Six months after the initial intervention, 45.2% of patients following IST and 45.9% of patients following IET had diabetes mellitus, either insulin-dependent or on oral medication (*p* = 0.946). During the same follow-up, a worsening of the diabetes mellitus level compared to the admission state was detected in 32.3% of the patients following IST and 31.1% of the patients following IET (*p* = 0.914). In long-term follow-up, the prevalence of diabetes mellitus was 50% after IST and 61.7% after IET (*p* = 0.281). Compared to the initial admission state, 37.5% of the patients after IST and 45.8% of the patients after IET showed a worsening of their diabetes mellitus. Six months after the initial intervention, the rate of exocrine insufficiency with dependence on oral medication (substitution of pancreatic enzymes) was 48.1% following IST and 21.3% following IET, which appeared to be statistically significant (*p* = 0.011). In long-term follow-up, 57.1% of the patients after IST and 16.4% of the patients after IET had persistent exocrine insufficiency (*p* =  < 0.001).

### Recurrence and survival

**Fig. 1:**
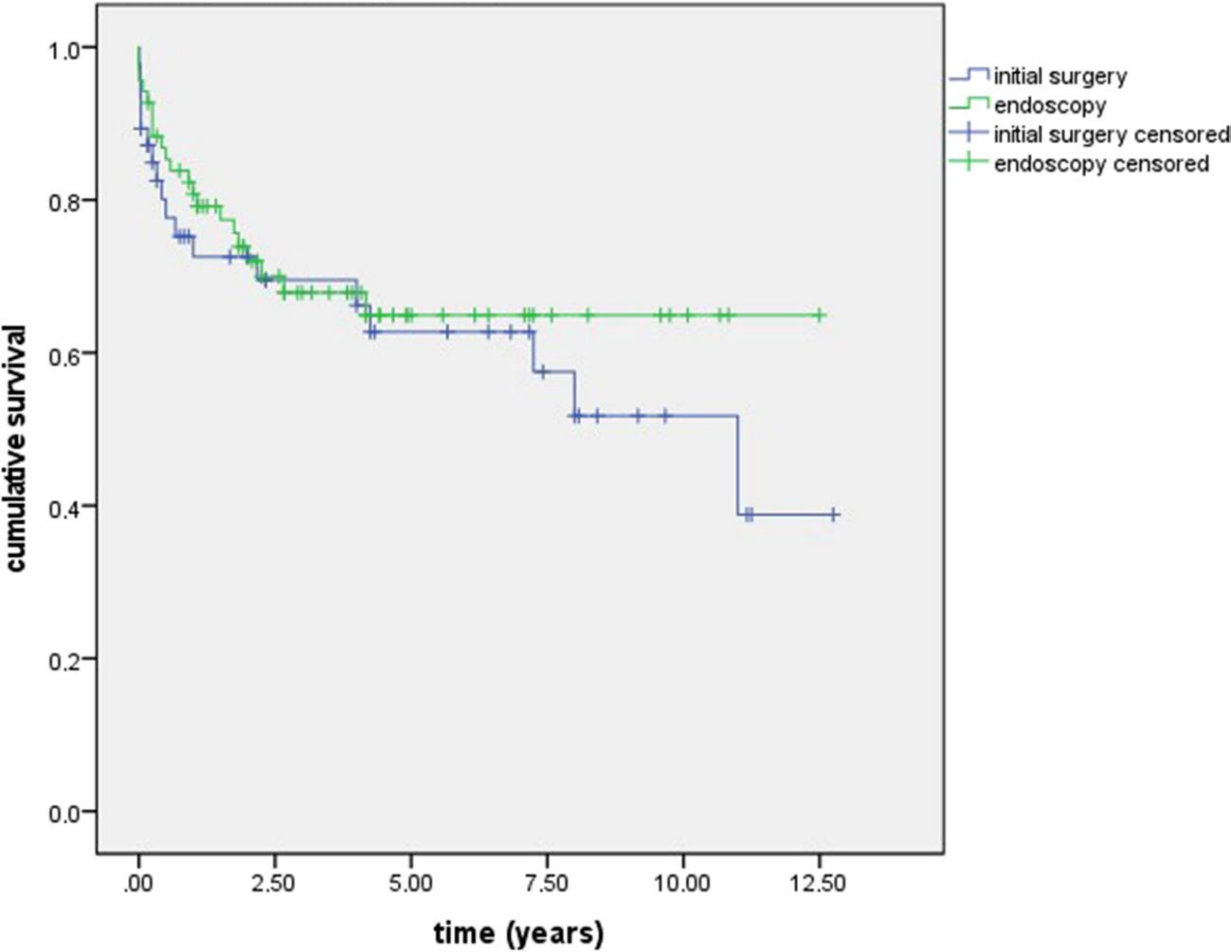
Overall survival function comparing patients undergoing either initial surgery or endoscopy

In the first 6 months, 4.9% of the patients following IET and 10.7% of the patients following IST showed recurrence of acute pancreatitis (*p* = 0.311). In the long-term follow-up, 8.9% of the patients following IET and 27.6% of the patients following IST showed recurrence of acute pancreatitis (*p* = 0.023). Seven patients after IET (10.1%) and ten patients after IST (22.2%) died during the initial in-hospital stay (*p* = 0.077). Following IST, the most common causes of death were severe septic shock (80%) or hemorrhagic shock (10%). Following IET, the most common causes of death were septic shock (43%), respiratory insufficiency (28%), and aspiration (14%). In the first 6 months of the follow-up, no additional patient from the IST group and three of the patients from the IET group died. One of them died due to pancreatic cancer, one due to bleeding of esophageal varices, and one due to pulmonary complications. In the long-term follow-up, the overall mortality was 45% for IST and 31.3% for IET (*p* = 0.156). Forty-three percent of the patients following IST died due to malignancy, 14% to pancreatitis, and 14% to respiratory complications. In the long-term follow-up of the IET patients, 56% died due to malignancy, 11% to ischemic stroke, 11% to cardiovascular diseases, and 11% to suicide. Figure 1 characterizes the overall survival function of either group.

## Discussion

In this study, we present our institutional short- and long-term results of patients being treated for acute necrotizing pancreatitis either by a surgery (IST)- or endoscopy (IET)-first concept. The in-house mortality in our cohort was higher in the surgical group, with 22.2%, compared to the endoscopic group, with 10.1%, although these findings did not appear statistically significant (*p* = 0.077). Although endoscopic treatment led to an overall reduction of mortality and morbidity in some studies [14, 15], van Brunschot et al. also showed comparable mortality rates for a surgical and an endoscopic approach [9]. Here, we need to underline that results from our and other retrospective, non-randomised studies comparing short-term mortality possibly underlie a fundamental selection bias as only patients with indication for emergency surgery or with inaccessible necrosis undergo initial surgery, whereas all other cases are treated with primarily endoscopic options. Ausania et al. have characterized it the “surgery effect” [16]. On the other hand, initial surgery might be judged as too invasive in elderly patients. The higher median age of the endoscopic group might be indicative for this hypothesis. In our cohort, the rates of pre-interventional sepsis, multi-organ failure, and renal insufficiency did not significantly differ. Only the amount of pre-interventional invasive ventilation was significantly higher in the surgical group. This could be due to our established concept, strictly adhering to the step-up approach, while even critically ill patients are treated with an endoscopic approach, when feasible. However, some indications still require emergency laparotomy, including intestinal perforation, severe bleeding, and abdominal compartment syndrome. Apart from these emergency indications, initial surgery is only indicated if the localization of the necrosis does not appear suitable for endoscopic drainage or minimal-invasive surgery. Nevertheless, the selection bias is not to be neglected as the indications for IST might predict greater damage to the pancreatic parenchyma on their own. Several studies showed a decrease in mortality if comparing initial surgery to endoscopic treatment in a step-up approach for acute necrotizing pancreatitis [15]. In our cohort, the endoscopic approach significantly reduced the overall in-hospital stay, the post-interventional in-hospital stay, the overall stay, and the post-interventional stay on a high dependency care unit, highlighting the economic effects of a step-up approach in addition to its medical benefits. Bang et al. also found the overall costs significantly lower for endoscopic treatment compared to even minimal-invasive surgery [17]. The post-interventional morbidity was significantly higher in the surgical group, with significantly higher amounts of post-interventional kidney failure and newly implemented invasive ventilation. The necessity of dialysis was comparable in both groups pre- and post-interventionally. Patients treated with initial surgery are more likely to develop single- or multi-organ failure than with a minimal-invasive approach [18]. In our cohort, both the rates of post-interventional sepsis and multi-organ failure were comparable in both groups.

During the post-interventional course, patients in the surgical group showed a worsening in their endocrine function compared to the endoscopic group, whereas the prevalence of diabetes mellitus did not significantly differ long-term. In the peri-interventional phase, the rate of exocrine dysfunction was higher in the surgical group, but these findings did not appear statistically significant. In the follow-up period after 6 months and the long-term follow-up, the rate of exocrine insufficiency in the surgical group was significantly higher, with 48.1% in the surgical group and 21.3% in the endoscopic group after 6 months and 57.1% of the patients in the surgical group and 16.4% in the endoscopic group during long-term observation, respectively. As the endocrine dysfunction appears to deteriorate in our cohort regardless of the approach, the exocrine dysfunction worsens following initial surgery, as it seems to recover following an endoscopic debridement. Connor et al. showed in a mean follow-up of 28.9 months following minimal-invasive or open necrosectomy that 25% of their patients developed exocrine and 33% endocrine insufficiency [19]. Onnekink et al. also compared the rate of endocrine and exocrine insufficiencies in the long-term follow-up of a randomized controlled trial and did not find significant differences between their surgical and endoscopic step-up approach [20]. As a randomized trial, the initial extent of necrosis and severity of pancreatitis was likely comparable in both of their groups. This may explain the differing results in our analysis. In long-term follow-up, 8.9% of the endoscopic and 27.6% of the surgical patients in our cohort showed recurrence of acute pancreatitis, which appeared to be statistically significant. Both findings might correlate, as the pancreatic enzymes have a role in the pathophysiology of acute pancreatitis. Additionally, a randomized trial found the systemic inflammatory response in the form of IL-6 levels to be significantly lower following endoscopic compared to surgical necrosectomy [21].

## Conclusion

The endoscopic approach appears favorable regarding morbidity and mortality and long-term preservation of the exocrine function, while no clear advantage on the endocrine function was observed. A step-up approach with close-knit interdisciplinary clinical evaluation is mandatory in order to decrease morbidity and mortality of this critical disease.

## Data Availability

No datasets were generated or analysed during the current study.
